# Network Biology Identifies Novel Regulators of CFTR Trafficking and Membrane Stability

**DOI:** 10.3389/fphar.2019.00619

**Published:** 2019-06-04

**Authors:** Cláudia Almeida Loureiro, João D. Santos, Ana Margarida Matos, Peter Jordan, Paulo Matos, Carlos M. Farinha, Francisco R. Pinto

**Affiliations:** ^1^BioISI—Biosystems & Integrative Sciences Institute, Faculty of Sciences, University of Lisbon, Lisbon, Portugal; ^2^Department of Human Genetics, National Health Institute “Dr. Ricardo Jorge,” Lisbon, Portugal; ^3^Department of Chemistry and Biochemistry, Faculty of Sciences, University of Lisbon, Lisbon, Portugal

**Keywords:** CFTR, interactome, membrane traffic, network biology, plasma membrane

## Abstract

In cystic fibrosis, the most common disease-causing mutation is F508del, which causes not only intracellular retention and degradation of CFTR, but also defective channel gating and decreased membrane stability of the small amount that reaches the plasma membrane (PM). Thus, pharmacological correction of mutant CFTR requires targeting of multiple cellular defects in order to achieve clinical benefit. Although small-molecule compounds have been identified and commercialized that can correct its folding or gating, an efficient retention of F508del CFTR at the PM has not yet been explored pharmacologically despite being recognized as a crucial factor for improving functional rescue of chloride transport. In ongoing efforts to determine the CFTR interactome at the PM, we used three complementary approaches: targeting proteins binding to tyrosine-phosphorylated CFTR, protein complexes involved in cAMP-mediated CFTR stabilization at the PM, and proteins selectively interacting at the PM with rescued F508del-CFTR but not wt-CFTR. Using co-immunoprecipitation or peptide–pull down strategies, we identified around 400 candidate proteins through sequencing of complex protein mixtures using the nano-LC Triple TOF MS technique. Key candidate proteins were validated for their robust interaction with CFTR-containing protein complexes and for their ability to modulate the amount of CFTR expressed at the cell surface of bronchial epithelial cells. Here, we describe how we explored the abovementioned experimental datasets to build a protein interaction network with the aim of identifying novel pharmacological targets to rescue CFTR function in cystic fibrosis (CF) patients. We identified and validated novel candidate proteins that were essential components of the network but not detected in previous proteomic analyses.

## Introduction

CFTR is a polytopic integral membrane protein that belongs to the ATP-binding cassette (ABC) transporter superfamily functioning as cAMP- and phosphorylation-regulated chloride and bicarbonate at the apical membrane of epithelial cells. Mutations in the *CFTR* gene cause cystic fibrosis, the most common autosomic recessive disorder among Caucasians (Riordan, 2008). The most prevalent CF-causing mutation is F508del, which is present in about 90% of CF patients and generates a mutant protein recognized by endoplasmic reticulum (ER) quality control (ERQC) mechanisms (Farinha and Amaral, 2005; Farinha et al., 2013; Farinha and Canato, 2017) and targeted for proteasomal degradation (Jensen et al., 1995).

Although most F508del-CFTR protein is only detected as a non-native (misfolded) core-glycosylated ER-specific intermediate, a small amount reaches the plasma membrane (PM) and is partially functional as a channel (Dalemans et al., 1991; Swiatecka-Urban et al., 2005). Thus, one important focus of basic research in CF involves attempts to rescue F508del-CFTR to the PM, by identifying either the key molecular factors involved in its ER retention or small molecules that promote its folding and release from the ERQC. Recent advances in the field allowed the identification of small molecules with potential to treat the basic defect in CF—either correctors, promoting the relocation of F508del-CFTR from the ER to the PM or potentiators, promoting channel activity (for variants already located at the PM). Among these, the potentiator VX-770 alone or its combinations with the correctors VX-809 or VX-661 made their way to the clinical practice, securing approval from the EMA and the FDA. However, results of these modulators in patients bearing F508del suggest that a physiologically relevant restoration of function will require additional small molecules with distinct corrective properties (Farinha and Matos, 2016). This may be due to the fact that F508del-CFTR, despite its partial pharmacological rescue of intracellular retention and impaired channel regulation, still presents decreased stability at the PM (Sharma et al., 2004; Swiatecka-Urban et al., 2005). Whereas the retention and the gating defects have been extensively studied, and both small-molecule correctors and potentiators are available, CFTR membrane stability is a less understood aspect, albeit crucial to restore F508del-CFTR to levels that correspond to clinical benefit.

CFTR traffic and activity at the PM is the result of numerous interactions that function as molecular switches (Amaral, 2005; Farinha et al., 2013) modulating channel activity and controlling the number of channel present at the membrane, through regulation of the secretory and membrane recycling pathways. This complex network of proteins is the last step in the control of the overall biogenesis/function of the protein and includes other transporters, channels, and trafficking machinery components (Rab GTPases, SNAREs, PDZ-domain-containing proteins) as well as different types of molecular switches (such as kinases, phosphatases, and small GTPases) (Guggino and Stanton, 2006; Farinha et al., 2013; Farinha and Canato, 2017).

Several mechanisms have been described to regulate CFTR anchoring and trafficking at the PM, which include CFTR phosphorylation by different kinases, interaction with cAMP sensors, or response to small GTPase signaling.

Phosphorylation in the regulatory region of CFTR is known for long to be required for its activity and it involves protein kinases A (PKA) and C (PKC) (Schultz et al., 1999). Together with PDZ-domain containing proteins, phosphorylation is responsible for the formation of multiprotein signaling complexes that provide spatial and temporal specificity to CFTR function (Guggino and Stanton, 2006). Besides this well-documented role on function, phosphorylation by spleen tyrosine kinase (Syk) in NBD1 (Luz et al., 2011; Mendes et al., 2011) or by lemur tyrosine kinase 2 LMTK2 in the R region (Luz et al., 2014) was shown to control the levels of CFTR at the PM.

cAMP levels in the cell lead to PKA activation and hence promote CFTR function, as described above. cAMP also activates members of guanine nucleotide-exchange factors, called EPACs (exchange proteins directly activated by cAMP), whose activation does not lead to PKA activation (Bos, 2006). Activation of EPAC1, the most abundant EPAC family member expressed in the lung, promotes its interaction with CFTR, leading to its stabilization at the PM through interaction with the PDZ-containing anchor protein NHERF-1 (Lobo et al., 2016).

Overexpression of NHERF1 promotes apical expression of the F508del-CFTR mutant channel (Guerra et al., 2005). This effect relies on the activation of endogenous RAC1 signaling and may also contribute to the increase of F508del-CFTR PM levels (Moniz et al., 2013; Loureiro et al., 2015).

Here, we used network biology to identify relevant protein nodes that may connect the interactomes obtained by analyzing different mechanisms regulating CFTR stability, namely, through phosphorylation by SYK or activation of EPAC1 or RAC1. We focused the network analysis on proteins never described to interact with or regulate CFTR and show that this approach is a valid strategy for finding novel regulators of CFTR function and traffic. Network biology thus proves to be a tool capable of contributing insights into the etiology of other human diseases linked to chloride transport or membrane protein traffic.

## Materials and Methods

### Protein Interaction Networks

Human physical protein–protein interaction data were extracted from HuRI (Human Reference Protein Interactome Mapping Project) (interactome.baderlab.org) (Rual et al., 2005; Venkatesan et al., 2009; Yu et al., 2011; Rolland et al., 2014; Yang et al., 2016) and APID (Agile Protein Interaction DataAnalyzer) (apid.dep.usal.es) (Alonso-Lopez et al., 2016) databases (accessed in June 2018). APID gathers literature-reported protein interactions, while HuRI results from unbiased large-scale screens for binary interactions. We constructed undirected and unweighted networks using Igraph R-package (Csardi and Nepusz, 2006). Loop and multiple edges were eliminated and only the main component of the network was selected. The network was further filtered to contain only proteins that are not exclusively located in nucleus, mitochondria, or peroxisomes. Gene ontology cellular component annotations obtained from UNIPROT were used to define protein subcellular location. This filter was necessary since the relevant interactions affecting CFTR stability at the PM should not occur within these three cellular compartments. The resulting operational network contained 17,218 proteins and 252,472 interactions.

### Network Analysis

Candidate proteins were selected through a neighbor enrichment test and a bridge score. The neighbor enrichment test evaluates if the set of direct neighbors of a candidate protein is enriched in members of an input protein set using a hypergeometric test. The bridging score quantifies for every node in a network its relevance in connecting two other nodes (provided as inputs) through short paths of length 2, 3, or 4. It counts the fraction of paths of length 2, 3, or 4 linking the two input nodes that contain a candidate node. These fractions for each length are used in a weighted sum where paths of length 2 have weight 1, paths of length 3 have weight 0.5, and paths of length 4 have weight 0.25. Both methods are implemented as R functions (*specific_neighbors* and *bridge_score*, respectively) publicly available in the NetSetStat repository (github.com/GamaPintoLab/NetSetStat). Proteins selected through both methods were analyzed for biological process enrichment using the DAVID tool (Huang Da et al., 2009a; Huang Da et al., 2009b) with the *Homo sapiens* genome as background and testing terms from the FAT subset of the Biological Process branch of Gene Ontology. Enriched GO terms were considered significant with a False Discovery Rate lower than 0.05 and were excluded if their background frequency was greater than 10% or lower than 5%.

### Preparation of siRNA-Coated 96-Well Plates

Two siRNAs against each of the following targets (CUL3, GABARAP, GABARAPL1, GABARAPL2, ILK, IQGAP1, LRRK2, NF2, NOS2, SMURF1, and UBASH3B) were acquired as a cherry-pick siGenome library (Dharmacon, Cambridge, UK); 96-well plates were coated with customized siRNAs for solid-phase reverse transfection. For that, a transfection mixture of 300 µl was prepared containing 161.5 µl of the Lipofectamine 2000 (Invitrogen #11668019) and 138.5 µl of 0.4 M sucrose in OptiMEM solution. Then, 1.25 µl of siRNA 20 µM was added per well into 96-conic well plates (siRNA plate); 1.75 µl of transfection mix was added per well to siRNA plate, followed by mixing and centrifugation for 1 min at 50×*g*. After 30 min incubation at room temperature, 1.75 µl of 0.2% (w/v) gelatin solution was added to the siRNA plate and centrifuged at 50×*g*. Then, the total of 4.25 µl of the mix in siRNA plate (siRNA plus transfection mix plus gelatin) was diluted 1:50 in a 96-conic well plate using double-distilled water. Finally, 50 µl from the diluted mix was lyophilized and stored in an anhydrous atmosphere before cell seeding.

### Reverse Transfection With siRNA

CFBE cells expressing an inducible doxycycline promotor for mCherry-Flag-CFTR (kind gift of Prof. Margarida Amaral, Lisbon) were grown to confluence and split. Then, after 24 h, cells were seeded in a siRNA-coated 96-well plate (100 µl of cell suspension/well, 7 × 10 ^7^ cells/well) using a Multidrop^™^ Combi Reagent Dispenser (Thermo Scientific #5840300). After 24 h of reverse transfection, CFTR expression was induced for 24 h for wt-CFTR and 48 h for F508del-CFTR, using 1 µg/ml doxycycline (Sigma #9891) in antibiotic-free medium.

### CFTR Trafficking Assay

The CFTR trafficking assay was performed as previously documented (Almaca et al., 2011; Botelho et al., 2015). This assay uses a double-tagged version of CFTR with mCherry at the N-terminus and FLAG at the fourth extracellular loop. Usage of the mCherry tag allows for quantification of the total amount of CFTR protein expressed by each individual cell. The Flag tag allows one to quantify CFTR that is exclusively localized at the cell surface by usage of an antibody applied extracellularly without cell permeabilization. Hence, 72 h after seeding and siRNA knockdown, cells were washed once with cold PBS^+/+^ (1× PBS supplemented with 0.9 mM CaCl_2_, 0.5 mM MgCl_2_, pH 7.2) using Tecan Hydrospeed^™^ and immunostained for the extracellular Flag-tag (in non-permeabilized cells) for 1 h at 4°C with 1:500 anti-Flag^®^ M2 monoclonal antibody (Sigma-Aldrich #F1804). Then, cells were washed three times in ice with cold PBS^+/+^ and incubated 20 min with 3% (w/v) paraformaldehyde (PFA) at 4°C. Cells were then washed three times in ice with cold PBS^+/+^ and incubated with anti-mouse Cy5 conjugated secondary antibody (Invitrogen, #A10524). Cells were washed three times in ice with cold PBS^+/+^ and incubated for 1 h with 1:5000 Hoechst 33342 solution (Sigma #B2261) for nucleus staining. Finally, cells were washed in ice with cold PBS^+/+^ and immersed in PBS^+/+^. All solutions were prepared in PBS^+/+^ and primary and secondary antibodies were diluted in PBS^+/+^ supplemented with 1% (v/v) BSA.

### Image Acquisition

Cell imaging was performed at room temperature with an inverted wide-field fluorescence microscope Leica DMI6000 equipped with a 12-bit 1,344 × 1,024 pixel resolution DFC360FX camera (Leica) and a 10× objective. Exposure times at maximum light brightness for Hoechst, mCherry, and Cy5 were 50, 1,300, and 8,000 ms, respectively. The Hoechst channel was used for contrast-based autofocus. The experiment was conducted in triplicate and five images per well were collected.

### Image Analysis

Automatic image analysis was performed using open-source software tools [cell image analysis software CellProfiler^1^ (Carpenter et al., 2006) and R^2^] and a pipeline to measure CFTR traffic efficiency developed previously (Botelho et al., 2015). Using this pipeline, the background subtraction was first applied to each image to correct the image illumination and background fluorescence. Then, a quality control was also applied for each image to exclude cells that do not significantly express CFTR, present abnormal morphology, or contain a significant number of saturated pixels. Finally, CFTR traffic was measured in each cell using the fluorescence quantification according to the following formula:

CFTR Traffic Efficiency=PM  CFTRTotal  CFTRCFTR Traffic Efficiency=Cy5  Integrated  FluorescencemCherry  Integrated  Fluorescence  (Formula 1)

CFTR traffic efficiency was calculated using the median CFTR traffic efficiency for all cells in the image. Using this pipeline, we also remove out-of-focus images, images with high background fluorescence, and images with a lower number of cells (less than 20 cells). Then, the average of CFTR traffic efficiency for all images in wells transfected with siRNAs, which passed the quality control (traffic efficiency test), was compared with the one measured under control conditions—siRNA EGFP (Traffic Efficiency_control_) according to the following formula:

$$\text{Deviation Score}=\frac{{\text{Traffic Efficiency}}_{\text{test}}-{\text{Traffic Efficiency}}_{\text{control}}}{2\times {\text{SEM}}_{\text{control}}}(\text{Formula }2)$$

The standard error of the mean for the Traffic Efficiency is given by the SEM_control_ recorded for siRNA EGFP. Deviation scores (DSs) that are positive or negative correspond to traffic enhancers or inhibitors, respectively. The DS equation normalizes the Traffic Efficiency measures to the mean and variation of the negative controls included in the same 96-well plate. This normalization facilitates the comparison between independent replicates, as all values are relative to the mean and standard error of the mean (SEM) of the negative controls in the respective plate. A DS of 1 is the double of the negative control SEM, which means that the latter has a normalized value of 0.5.

### Analysis of siRNA Knock-Down Efficiency

CFBE cells constitutively expressing F508del-CFTR were seeded in 96-well dishes and transfected with the panel of siRNAs described above (Dharmacon, Cambridge, UK), using Lipofectamine 2000 (Invitrogen). All cells were incubated for 48 h at 37°C with 5% CO_2_ and then RNA was extracted with the NucleoSpin RNA kit (Macherey Nagel, Düren, Germany). For cDNA synthesis, 0.5 μg of total RNA was then reverse transcribed using random primers (Invitrogen/Life Technologies) and Ready-to-Go You-Prime beads (GE Healthcare, Buckinghamshire, UK). Real-time PCR quanti?cation (qPCR) was performed as previously described (Matos et al., 2008). Primers used in qPCR for each of the targeted transcripts are described in **Table 1**. Each cDNA sample was diluted fivefold to guarantee accurate pipetting and 5 μl was added to each real-time reaction together with 250 nM primers and SYBR Green Master Mix (Applied Biosystems/Life Technologies). Data were analyzed with the ABI Prism 7000 SDS 1.1 RQ Software (ΔΔCT method, Applied Biosystems/Life Technologies).

**Table 1:** Primers used for qPCR quantification of siRNA efficiency.

Gene name	Transcript_id	Forward primer sequence	Reverse primer sequence
CUL3	NM_003590.4	ACAGCTATGGTGATGATTAGAGAC	TACGACTTCTCCTTTCCGCT
GABARAP	NM_007278.1	GGCTCCCAAAGCTCGGATAG	GCAGCTTCACAGACCGTAGA
GABARAPL1	NM_001363598	TGCCTGATCTGGACAAGAGG	AACGCATCTAGAACAAGGGCT
GABARAPL2	NM_007285.6	TGTTCAAGGAGGACCACTCG	CCACAAACAGGAAGATCGCC
ILK	NM_004517.3	GCTTGGGGTTCATCCTCCTT	TTACATTGATCCGTGCCCCC
IQGAP1?	NM_003870.3	GCACTGGCTAAGACGGAAGT	CGGATAGCACGTCTCTGCAT
LRRK2	XM_005268629.4	GGCCCTCCTCACTGAGACTA	TGCATCAGCATGGAGAGCAT
NF2	NM_181832.2	AGAGGAGCTGGTTCAGGAGA	GCCAAAAATCCCCGCTTGTG
NOS2	NM_000625.4	CCTCGCTCTGGAAAGACCAG	GGGACAGGACGTAGTTCAGC
SMURF1	NM_181349.2	CGTGGGGAAGAAGGTTTGGA	AAGCCCCCGTTGATGTAGTG
UBASH3B	NM_032873.4	TGGACGTGCTCCTCTCCAT	GGGGAAGATGTTGTGTGCCT

### CFTR Functional Assay by Halide-Sensitive YFP (HS-YFP)

CFBE cells constitutively expressing wt- or F508del-CFTR together with HS-YFP-F46L/H148Q/I152L (Galietta et al., 2001) were seeded in eight-well chamber slides. F508del-CFTR cells were seeded in duplicate slides. Cells were then transfected with either siLUC (Eurofins MWG Operon), siGABARAP_1, siSMURF1_2, or siENOS_2 (Dharmacon) using Lipofectamine 2000 (Invitrogen). All cells were incubated for 48 h and VX-809 (3 μM; Gentaur) was added to one of the F508del-CFTR cell sets. Cells were carefully washed twice with isomolar PBS (WPBS: 137 mM NaCl, 2.7 mM KCl, 0.7 mM CaCl_2_, 1.1 mM MgCl_2_, 1.5 mM KH_2_PO_4_, 8.1 mM Na_2_HPO_4_, pH 7.4), and wt-CFTR cells were incubated for 15 min in WPBS containing 1 μM indomethacin to reduce endogenous cAMP levels. Cells were then transferred to a Leica TCS-SPE confocal microscope for time-lapse analysis. Each well was assayed individually for iodide influx by recording fluorescence continuously (500 ms per point), first for 10 s (baseline) and then for 60 s after the rapid (≤1 s) addition of isomolar PBS, containing either 5 μM Forskolin (Fsk) (wt-CFTR cells) or 5 μM Fsk plus 20 μM Gen (F508del-CFTR cells), in which 137 mM Cl^−^ was replaced by I^−^ (IPBS; final NaI concentration of 100 mM/plate well). Cells were kept at 37°C up until being assayed at room temperature. After background subtraction, HS-YFP fluorescence recordings (F) were normalized to the initial average value measured before addition of I^−^ (F_0_). Quantification of fluorescence decay was performed on at least 24 individual cells per well, using ImageJ (NIH) as previously described (Matos et al., 2018). The average fluorescence decay was fitted to an exponential decay function to derive the maximal slope [d(*F*/*F*_0_)/d*t*, at *t* = 0] that is proportional to the initial influx of I^−^ into the cells. (Loureiro et al., 2015).

### Protein Thermal Destabilization Assay (Thermal Shift Assay, TS)

After Dox-induced CFTR expression (1 μg/ml; Sigma-Aldrich), mCherry-Flag-F508del-CFTR CFBE cells were transfected with either siLUC (Eurofins MWG Operon) or siGABARAP_1 (Dharmacon), using Lipofectamine 2000. Cells were then incubated for 24 h with VX-809 (3 μM; Gentaur) at 37°C and then moved for another 24 h to 30°C, to increase the thermal stability of rescued F508del-CFTR at the PM and allow its accumulation at the cell surface. For the thermal shift assay, cells were again transferred to 37°C for 4 h to induce F508del-CFTR thermal destabilization and PM removal, as described previously (Okiyoneda et al., 2010). Cells were then placed on ice, washed three times with ice-cold WPBS, and left 5 min in cold WPBS to fully arrest CFTR trafficking. Afterwards, they were incubated on ice with anti-Flag M2 Ab (F3165, Sigma-Aldrich) in WPBS for 1 h at 4°C, without permeabilization. Cells were washed three times with ice-cold WPBS and incubated with anti-mouse Alexa Fluor 488 secondary Ab (Thermo Fisher Scientific) for 1 h at 4°C. Cells were then fixed with 4% formaldehyde for 15 min, thoroughly washed in PBS containing DAPI (4′,6-diamidino-2-phenylindole, Sigma-Aldrich), mounted on microscope slides with Vectashield (Vector Laboratories), and analyzed by confocal microscopy.

### Confocal Microscopy Imaging

Slides of mCherry-Flag-F508del-CFTR CFBE cells immunolabeled with anti-Flag followed by Alexa Fluor 488 were analyzed by confocal microscopy. Images were acquired on a Leica TCS-SPE confocal microscope, where mCherry fluorescence is proportional to the total amount of CFTR, Alexa Fluor 488 fluorescence is proportional to the amount of CFTR present at the cell surface, and nuclei were stained with DAPI. A 0.2-μm Z-stack of 1-Airy confocal images was acquired to select the best *XY* plane to analyze the CFTR protein at the cell surface. ImageJ software (NIH) was then used to delimitate each single cell as individual ROIs (regions of interest, to allow measurement of mCherry and Alexa488 fluorescence signal intensities). Quantification of fluorescence was performed on at least 24 individual cells per field, as previously described (Matos et al., 2018), and expressed as mean ± SEM fold variation over control cells.

### Statistical Analysis

*Multiple treatment data analysis*—Statistical significance of the observed variations between different treatments was assessed using two-tailed Kruskal–Wallis tests, followed by paired comparisons using the Mann–Whitney *U* test, considering a 0.05 significance level for both tests. All observations were confirmed in at least three independent experiments, each with two replicates for each individual condition tested.

*Statistical analysis of CFTR trafficking assay*—Each siRNA was used to measure traffic efficiency of wild-type CFTR, F508del-CFTR, and F508del-CFTR rescued with VX-809. For each siRNA and CFTR type, three independent replicate DSs were obtained. The DS mean was considered a measure of the siRNA capacity to influence the traffic efficiency of a particular CFTR type (<DS_i_>, i corresponding to CFTR type), and the sum of the three DS mean absolute values (Σ|<DS_i_>|) was considered a global measure of the siRNA effect. To evaluate statistical significance of these effects, a random permutation test was performed, where the data matrix containing all the individual DS (all replicates for all CFTR types and all siRNAs, including siRNAs without knockdown effect, functioning as additional controls) was randomly shuffled 10,000 times. Full matrix permutation is possible because all DS values are normalized by EGFP control traffic efficiency mean and SEM values. For each random matrix, each siRNA random Σ|<DS_i_>| was computed, generating distributions according to the null hypothesis that siRNAs have no effect. Null hypotheses were rejected when the fraction of random values greater than or equal to the observed Σ|<DS_i_>| was lower than 0.05.

## Results

### Network Connectivity between CFTR Interactors Potentially Modulating Stability at the PM

Studies from our team have identified five sets of CFTR interactors through three parallel approaches (under consideration elsewhere): a) proteins selectively interacting at the PM with either wt-CFTR (set 1: wt) or rescued F508del-CFTR (set 2: rescued), b) protein complexes involved in cAMP-mediated CFTR stabilization (set 3: EPAC), and c) adaptor proteins binding to tyrosine-phosphorylated (set 4: pep-py) or un-phosphorylated (set 5: pep-wt) CFTR peptide (unpublished data). All these complementary approaches were designed to identify potential modulators of CFTR PM stability through different pathways. We hypothesized that proteins simultaneously interacting with these different pathways may also have a significant influence on CFTR stability at the PM.

First, we mapped these sets of interactors on a human protein physical interaction network and explored how strongly they were connected through this network (**Table 2**). To evaluate the statistical significance of network connectivity, we had to consider that these interactor sets were selected by their presence in immunoprecipitated CFTR complexes. Therefore, we generated 500 random sets of interactors (for each of the five observed sets), with the same number of proteins at each observed network distance from CFTR. For each connectivity property (number of overlapping proteins between sets, number of direct interactions between set members, and number of common direct neighbors between sets), we recorded the average value across the 500 randomizations and a *p* value, which corresponds to the fraction of random set pairs showing a value higher than that observed with the experimental sets. The five sets have a low number of proteins in common, although 8 out of 10 pairs have a significantly higher overlap than expected by chance. The strong connectivity between these CFTR interactor sets is much more evident by looking at the numbers of direct physical interactions and common neighbors that are much higher than randomly expected and reach statistical significance for all set pairs. This strong connectivity supports our attempt to find new proteins simultaneously interacting with multiple CFTR PM stability-related pathways.

**Table 2:** Overlap, direct interactions, and common neighbors between CFTR interactor sets.

CFTR interactor sets		Overlap		Direct interactions		Common neighbors	
		Observed (expected)	*p*	Observed (expected)	*p*	Observed (expected)	*p*
wt	rescued	7 (1.2)	<0.002	183 (48.1)	<0.002	2,092 (1,244)	<0.002
	EPAC	3 (0.4)	0.004	54 (16.9)	<0.002	1,138 (692)	0.006
	pep-py	2 (0.3)	0.024	82 (15.3)	<0.002	1,535 (661)	<0.002
	pep-wt	0 (0.2)	0.547	28 (5.6)	<0.002	888 (310)	0.002
Rescued	EPAC	8 (1.1)	<0.002	164 (44.8)	<0.002	1,818 (1,191)	0.006
	pep-py	6 (0.9)	0.002	359 (41.8)	<0.002	2,300 (1,153)	<0.002
	pep-wt	3 (0.3)	0.002	113 (15.3)	<0.002	1,364 (515)	<0.002
EPAC	pep-py	1 (0.2)	0.117	57 (14.0)	<0.002	1,140 (636)	0.002
	pep-wt	2 (0.1)	0.002	23 (5.2)	<0.002	758 (301)	0.008
pep-py	pep-wt	4 (0.1)	<0.002	94 (5.0)	<0.002	1,473 (294)	<0.002

### Specific Common Neighbors of CFTR Interactor Sets

**Figure 1:**
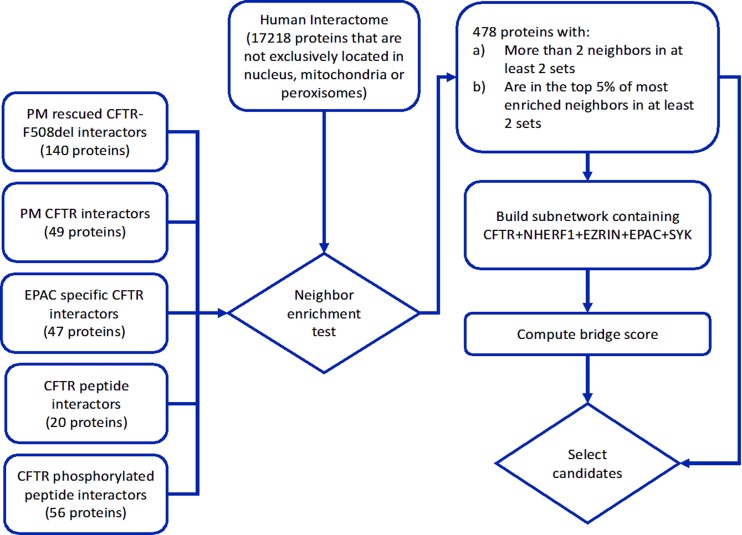
Diagram of the network analysis procedures. CFTR interactor sets were mapped on a human protein physical interaction network, filtered for protein subcellular location to guarantee plausible interactions with CFTR. Proteins in the network were submitted to a neighbor enrichment test to select specific common neighbors of the CFTR interactor sets. A subnetwork containing the selected specific neighbors, CFTR, and related stability factors (EPAC, NHERF1, EZRIN, and SYK) was assembled and a bridge score was computed to identify strong network mediators of CFTR interaction with the referred stability factors. The ranking of neighbor specificity and bridge score were used to select candidates for experimental validation of CFTR PM stability modulation.

We have identified 4411 proteins that have direct physical interactions with proteins in at least two CFTR interactor sets (2,420, 1,161, and 443 with direct neighbors in at least 3, 4, and 5 sets, respectively). However, to increase the likelihood that perturbations in one of these proteins will have an effect in these specific neighbor pathways and, consequently, on CFTR PM stability, we should require that these proteins are enriched in direct interactions with these CFTR interactor sets. With that aim, we performed a hypergeometric test to determine if these proteins are enriched in interactions with each of the five sets. As we performed this test to all proteins with neighbors in each of the five interactor sets, choosing a trivial *p* value cutoff may be associated with many false positives. To avoid this multiple testing problem, we selected for each set of CFTR interactors the 5% proteins with the lowest hypergeometric *p* values as specific neighbors of that set, and additionally required the selected proteins to have at least three interactions with two or more CFTR interactor sets. This strategy resulted in the selection of 478 proteins. We ranked these proteins by summing the ranks of their neighbor enrichment *p* values for all sets, discarding sets where the protein was not in the top 5% of significantly enriched proteins (protein list and associated statistics are available in **Supplementary Table 1** and the selection process is depicted in **Figure 1**).

### Important Mediators of CFTR Interaction With EPAC, NHERF1, EZRIN, and SYK

The CFTR interactor sets were experimentally detected under conditions that modulate CFTR PM stability through the influence of factors such as EPAC, NHERF1, EZRIN, and SYK, previously known to be involved in the stabilization process (Mendes et al., 2011; Loureiro et al., 2015; Lobo et al., 2016). To uncover novel proteins that most likely influence CFTR PM stability, we aimed to quantify the contribution of a given protein to interfere (either positively or negatively) with the formation of complexes including CFTR and one of the referred stability factors.

For this purpose, we developed a bridge score that measures the fraction of short length paths (with two to four interactions) connecting two proteins of interest (CFTR and the stability factor) that are mediated by a candidate protein. Mediation of shorter paths is given a higher weight/score. The perturbation of proteins with high bridge scores will disrupt a higher fraction of short network paths linking the two proteins of interest. If the interactions in these paths stabilize or facilitate the assembly of the complex containing the two proteins of interest, the bridging protein may have a positive effect on the functional interaction between the two proteins. Reversely, if the interactions in the short paths compete with the formation of the complex, the bridging protein may negatively affect the functional interaction between the two proteins.

To compute the bridge score, we assembled a connected subnetwork containing CFTR, the four stability factors, and the common neighbors identified in the previous section (process described in **Figure 1**). The resulting subnetwork contained 482 proteins and 16,481 interactions. We considered a protein to be an important mediator between CFTR and one stability factor if its bridge score was in the top 25% of all scores with the corresponding stability factor. This procedure allowed the identification of 194 strong mediator proteins (**Supplementary Table 1**). Gene ontology analysis (**Supplementary Table 2**) showed that the two biological processes most significantly enriched within these 194 proteins were “protein localization to organelle” (GO:0033365, 54 proteins) and “regulation of protein modification process” (GO:0031399, 71 proteins), which supports their hypothetical role in the modulation of CFTR PM stability.

### Hit Selection

To assess the impact of protein networks in CFTR trafficking, we selected hits for validation from the list of 478 proteins significantly enriched in neighbors from at least two CFTR interactor sets.

From the list of specific common neighbors of CFTR interactors, we started by excluding any proteins detected in the three initial experimentally derived interactor sets. Then, we focused on the top 25% of the list (proteins with higher neighbor enrichment rank sum as defined above). Then, we used data from Human Protein Atlas and excluded proteins with low expression in the “bronchus” (keeping thus only those with medium, high, or n/a protein expression). As we were interested in assessing CFTR PM stability, we then excluded proteins with annotation as ribosomal proteins/elongation or transcription factors/HNRNP/RNA processing/nuclear proteins. The next step was to exclude proteins already detected as CFTR interactors in previous studies (Wang et al., 2006; Pankow et al., 2015). This refined our list from 478 to 44 proteins. Finally, we used literature mining and refined this list to 25%, selecting 11 possible hits for validating the proteins listed in **Table 3**.

**Table 3:** Hits selected for validation.

UniProt identifier	Gene	Protein	Specific neighbor of	Strong mediator of CFTR with
Q9H0R8	GABARAPL1	Gamma-aminobutyric acid receptor-associated protein-like 1 (Early estrogen-regulated protein) (GABA(A) receptor-associated protein-like 1) (Glandular epithelial cell protein 1) (GEC-1)	All sets	EZRIN, NHERF1, SYK
O95166	GABARAP	Gamma-aminobutyric acid receptor-associated protein (GABA(A) receptor-associated protein) (MM46)	All sets	NHERF1
P60520	GABARAPL2	Gamma-aminobutyric acid receptor-associated protein-like 2 (Early estrogen-regulated protein) (GABA(A) receptor-associated protein-like 2) (Glandular epithelial cell protein 2)	All sets	EPAC, EZRIN, NHERF1, SYK
P35228	NOS2	Nitric oxide synthase, inducible (EC 1.14.13.39) (Hepatocyte NOS) (HEP-NOS) (Inducible NO synthase) (Inducible NOS) (iNOS) (NOS type II) (Peptidyl-cysteine *S*-nitrosylase NOS2)	All sets	NHERF1
Q5S007	LRRK2	Leucine-rich repeat serine/threonine-protein kinase 2 (EC 2.7.11.1) (Dardarin)	All sets	EPAC, EZRIN, NHERF1, SYK
P35240	NF2	Merlin (Moesin-ezrin-radixin-like protein) (Neurofibromin-2) (Schwannomerlin) (Schwannomin)	All sets	EZRIN, NHERF1
Q13418	ILK	Integrin-linked protein kinase (EC 2.7.11.1) (59 kDa serine/threonine-protein kinase) (ILK-1) (ILK-2) (p59ILK)	All sets except pep-wt	
Q9HCE7	SMURF1	E3 ubiquitin-protein ligase SMURF1 (hSMURF1) (EC 2.3.2.26) (HECT-type E3 ubiquitin transferase SMURF1) (SMAD ubiquitination regulatory factor 1) (SMAD-specific E3 ubiquitin-protein ligase 1)	All sets except wt	NHERF1, SYK
Q8TF42	UBASH3B	Ubiquitin-associated and SH3 domain-containing protein B (EC 3.1.3.48) (Cbl-interacting protein p70) (Suppressor of T-cell receptor signaling 1) (STS-1) (T-cell ubiquitin ligand 2) (TULA-2) (Tyrosine-protein phosphatase STS1/TULA2)	All sets except wt	SYK
Q13618	CUL3	Cullin-3 (CUL-3)	All sets	EZRIN, NHERF1, SYK
P46940	IQGAP1	Ras GTPase-activating-like protein IQGAP1 (p195)	All sets except pep-py	EZRIN, NHERF1, SYK

### Assessment of CFTR Trafficking Under Knockdown of Selected Hits

We ordered two different siRNAs for each of the putative hit proteins from Dharmacon’s siGenome library (see Materials and methods). We then used qPCR to assess the knockdown efficiency of the targeted transcript by each siRNA in CFBE cells (**Figure S1**). We verified that most siRNA pairs exhibited considerable differences in their ability to knock down the abundance of the mRNAs coding for the targeted proteins. Notwithstanding, we observed that the siRNAs GABARAP_1, GABARAPL2_1, IQGAP1_1, LRRK2_2, NF2_2, NOS2_2, and SMURF1_2 were able to significantly (*p* < 0.05) downregulate the hit mRNAs that they targeted (**Figure S1**), and these were chosen for further analysis on CFTR trafficking (wt-, F508del-, and F508del-CFTR rescued with VX-809). We used CFBE cell lines stably expressing a double-tagged (mCherry and FLAG) version of CFTR under the control of a doxycycline-inducible promoter so that CFTR expression could be induced 24 h after transfection with siRNA and immunofluorescence be performed at 24 h post-induction. CFTR at the PM is monitored by the Flag (Cy5) signal and total CFTR by mCherry fluorescence. CFTR trafficking is given by the ratio of PM CFTR to total CFTR (Botelho et al., 2015).

For this assay, cells were also transfected with two previously validated siRNAs targeting CFTR and COPBI (Botelho et al. 2015), as controls of the transfection efficiency and trafficking inhibition, respectively.

**Figure 2:**
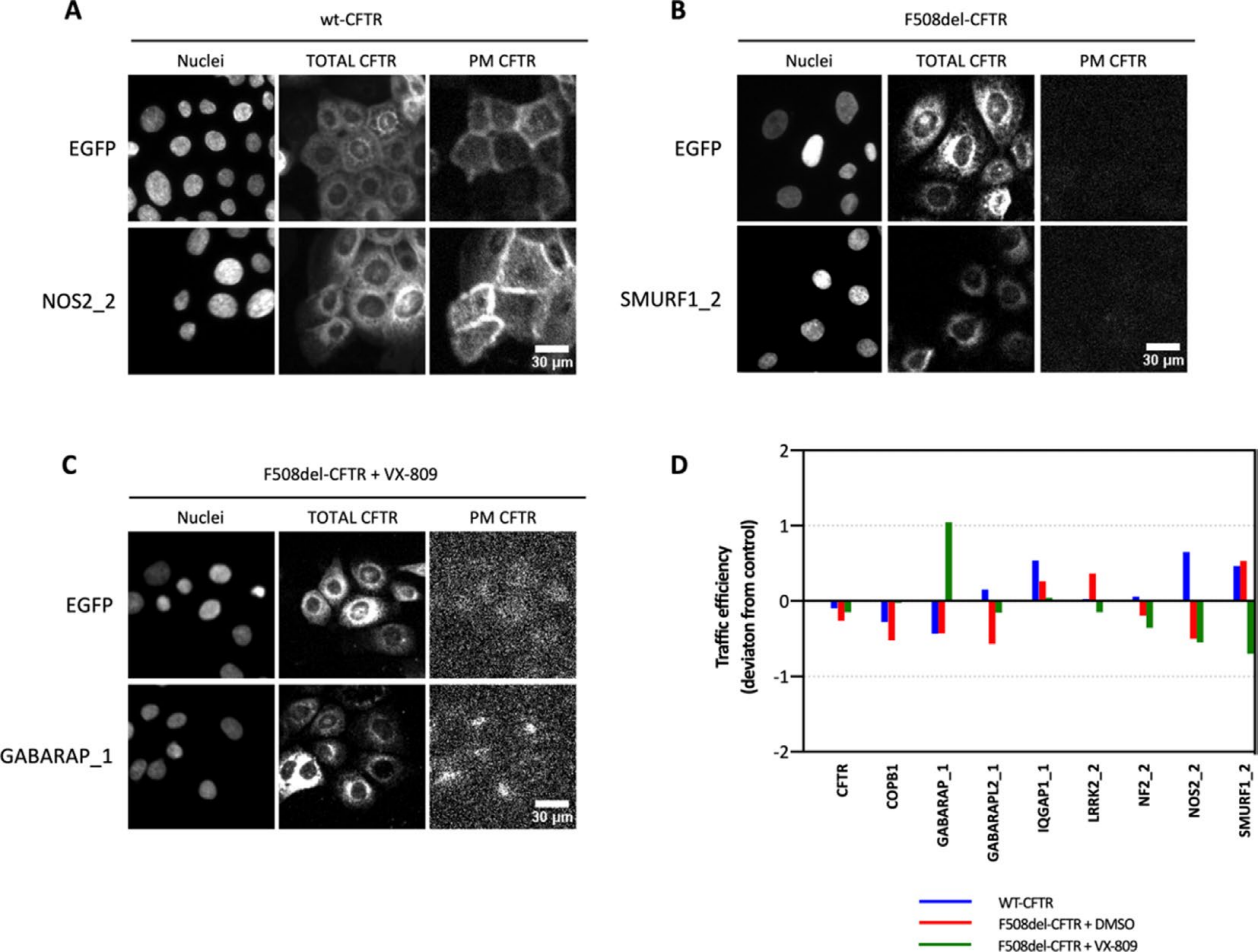
CFTR trafficking upon knock-down of the possible hits. CFBE cell lines expressing mCherry-Flag-wt-, mCherry-Flag-F508del-, and mCherry-Flag-F508del-CFTR treated with VX-809 (3 μM for 48 h) under the control of a doxycycline-inducible promoter were transfected with siRNAs targeting CUL3, GABARAP, GABARAPL1, GABARAPL2, ILK, IQGAP1, LRRK2, NF2, NOS2, SMURF1, and UBASH3B. NT (non-treated cells) and COPB1 and EGFP (no-target siRNA) siRNA were used as controls. **(A–C)** Representative images for the three cell lines and one target for each cell line. PM CFTR image contrast was optimized after quantification to allow visualization of detected signal differences. **(D)** The CFTR traffic efficiency is obtained with the ratio PM/total CFTR with PM CFTR assessed by Flag staining (Cy5 fluorescence) and the amount of total CFTR with the mCherry signal. Data are shown as the deviation score as detailed in Formula 2, where positive values correspond to traffic efficiencies larger than the negative control (siRNA against EGFP) and negative values correspond to traffic efficiencies lower than the negative control, *n* = 3.

Results showed that the different proteins affected differently the three CFTR conditions tested (**Figure 2**). **Table 4** summarizes the top seven siRNAs that, when transfected into the three cell lines, promoted the largest increases in CFTR trafficking efficiency.

**Table 4:** Deviation score (DS)-based analysis of CFTR traffic efficiency for wt- or F508del-CFTR (incubated with either DMSO or VX-809) following the siRNA-mediated hit protein knockdown.

siRNA	<DS_wt_>	<DS_F508del_>	<DS_F508del+VX-809_>	∑|<DS_i_>|	*p*
GABARAP_1	−0.433	−0.431	1.049	1.912	**0.013**
GABARAPL2_1	0.151	−0.570	−0.153	0.875	0.449
IQGAP1_1	0.536	0.264	0.044	0.844	0.479
LRRK2_2	0.031	0.364	−0.146	0.540	0.794
NF2_1	−0.701	0.319	−0.761	1.781	**0.023**
NOS2_2	0.650	−0.501	−0.549	1.701	**0.031**
SMURF1_2	0.463	0.533	−0.698	1.693	**0.032**

DS_i_ (i corresponding to wt-, F508del-, or F508del-CFTR+ VX-809) informs on the siRNA capacity to influence the traffic efficiency of a particular CFTR type. The sum of the three DS mean absolute values (∑|<DS_i_>|) constitutes a global measure of the siRNA effect. Statistical significance (*p*) of these effects were calculated using a random permutation test (see Materials and Methods for details). Values highlighted in bold when significant.

Altogether, we observed that siRNAs GABARAP_1, NOS2_2, and SMURF1_2 elicited consistent increments in the trafficking and overall PM levels of VX-809-rescued F508del-, wt-, and untreated F508del-CFTR, respectively (**Figure 2**), prompting us to analyze their impact on overall CFTR function in CFBE cells.

### Knockdown of GABARAP and NOS2 Modulates wt- and VX-809 Rescued F508del-CFTR Function in Opposite Ways

**Figure 3:**
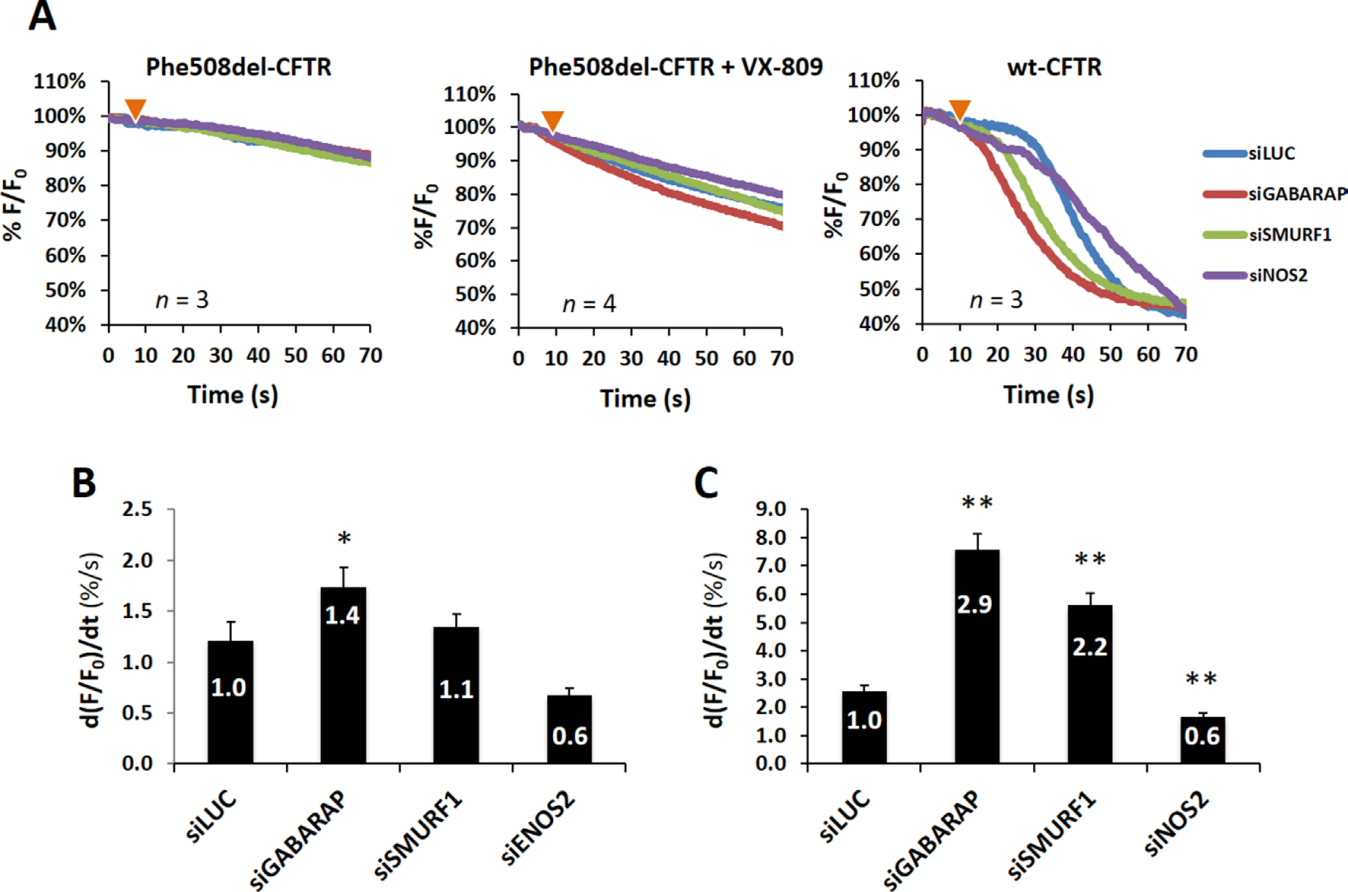
Knockdown of selected candidate proteins influences wt- and F508del-CFTR function. **(A)** Representative fluorescence decay curves of the iodide influx assay. Wt- or F508del-CFBE cells stably expressing the HS-YFP sensor were transfected and treated for 48 h, as indicated. Fluorescence microscopy images were acquired continuously, first for 10 s (baseline) and then for 60 s after the rapid (≤1 s) addition of isosmotic buffer, containing 100 mM iodide together with either 5 μM Fsk (wt-CFTR cells) or 5 μM Fsk plus 20 μM Gen (F508del-CFTR cells). **(B)** Iodide influx rates from VX-809-treated (3 μM, 48 h) F508del-CFTR cells, transfected with the indicated siRNAs. The average fluorescence decay was fitted to an exponential decay function to derive the maximal slope [d(*F*/*F*_0_)/d*t*, at *t* = 0] that is proportional to the initial influx of I^−^ into the cells. **(C)** Iodide influx rates, calculated as in **(B)**, for wt-CFTR CFBE cells, transfected as indicated. Data are means ± SEM of three independent assays. *p < 0.05; **p < 0.01.

To test the effect of knocking down the expression of the three selected candidates on CFTR function, we used a widely used and previously validated functional assay, based on the ability of iodide to quench the fluorescence of the halide-sensitive YFP-F46L/H148Q/I152L mutant (HS-YFP) (Loureiro et al., 2015; Matos et al., 2018). As shown in **Figure 3**, knockdown of either SMURF1, NOS2, or GABARAP had no apparent effect on HS-YFP fluorescence in untreated F508del-CFTR cells, compared to control cells (siLUC). In contrast, following VX-809 treatment of cells to allow some F508del-CFTR protein to reach the PM, the downregulation of GABARAP produced a moderate but clear effect on HS-YFP quenching (**Figure 3A**), corresponding to a significant (*p* < 0.05) 1.4-fold increase in the initial iodide influx rate, as calculated from the exponential decay fit of quantified fluorescence values (**Figure 3A**). Interestingly, whereas the downregulation of either SMURF1 or NOS2 appeared to have no significant effect on rescued F508del-CFTR, the knockdown of NOS2 produced an apparent 40% decrease in influx rate, when compared to control (siLUC) cells (**Figure 3B**). Notably, similar and statistically significant opposite effects were observed upon the downregulation of GABARAP and NOS2 in wt-CFTR cells (**Figure 3A and C**). Moreover, despite showing no observable consequence in the trafficking assay, the increment produced by GABARAP knockdown in these cells was clearly pronounced, triggering a 2.9-fold faster CFTR response, even after endogenous cAMP reduction by pretreatment of the cells with 1 μM indomethacin, prior to stimulation with 5 μM Fsk. Also noteworthy, the knockdown of SMURF1, which failed to produce a clear increase in wt-CFTR PM signal in the trafficking assay, produced a significant (*p* < 0.01) over twofold stimulation of the iodide influx rate in these cells (**Figure 3C**). Overall, interference with either of the three candidate proteins interfered with functional activity of wt-CFTR, but only GABARAP and NOS2 affected the clinically relevant rescued F508delCFTR, producing opposite functional outputs.

### GABARAP Knockdown Increases the PM Stability of VX809-Rescued F508del-CFTR

**Figure 4:**
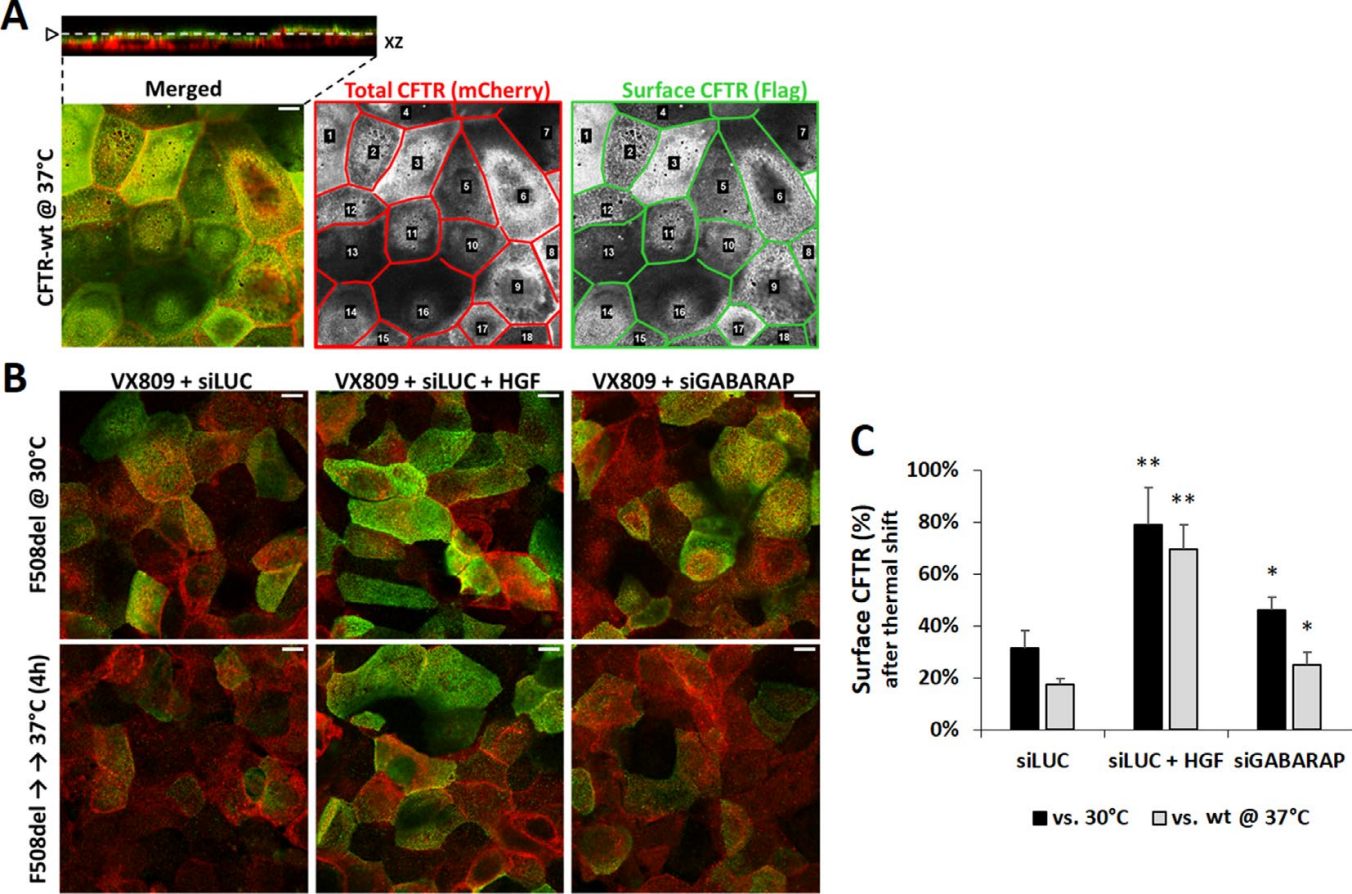
GABARAP downregulation increases rescued F508del-CFTR PM abundance. **(A)** Confocal images of Dox-induced, mCherry-Flag-wt-CFTR expressing CFBE cells. Images are of double-labeled non-permeabilized cells, where mCherry fluorescence is proportional to the total amount of CFTR and Alexa Fluor 488 fluorescence is proportional to the amount of CFTR present at the cell surface. A 0.2-μm Z-stack of 1-Airy confocal images was acquired (top left inset) to select the best *XY* plane to analyze the CFTR protein at the cell surface. ImageJ software (NIH) was used to delimit each single cell as individual ROIs (regions of interest) to allow measurement of mCherry (red lined lattice) and Alexa488 (green lined lattice) fluorescence signal intensities. **(B)** Confocal images, obtained as in (A) of Dox-induced, VX-809-treated (3 μM, 48 h) mCherry-Flag-F508del-CFTR CFBE cells, transfected with either siLUC or siGABARAP and co-treated with 50 ng/ml HGF, when indicated. Cells were first pre-incubated for 24 h at 30°C to stabilize PM rescued F508del-CFTR and then moved to 37°C for 4 h to allow destabilization and internalization of the recued protein, unless otherwise prevented by the treatment conditions. White bars correspond to 10 μm. **(C)** Analysis of single-cell fluorescence signals in three independent experiments. Data presented refer to mean values ± SEM of three independent assays. CFTR at the surface was estimated by the ratio of Flag/mCherry signals in all conditions and expressed as percentage of surface F508del-CFTR at 30°C (siLUC, black bars) or surface wt-CFTR at 37°C (gray bars). *p < 0.05; **p < 0.01.

Because the absolute increment of VX-809-rescued F508del-CFTR at the cell surface exceeded that computed for its trafficking efficiency upon downregulation of GABARAP, we asked whether the observed increase in both CFTR PM abundance and ion transport would rather reflect an improved stability of the rescued channels at the PM. To test this, we used the previously described thermal shift assay (Loureiro et al., 2015). This assay employs the mCherry-Flag-F508del-CFTR CFBE cell line used for the trafficking assay but with a different experimental procedure. First, F508del-CFTR is rescued with VX-809 and then stabilized at the cell surface by incubating the cells at 30°C for 24 h. Next, cells are returned to 37°C for 4 h, which destabilizes the rescued mutant channels causing their endocytic removal from the cell surface, unless the tested co-treatment procedure is able to delay or prevent CFTR internalization. Cells are then stained as before and analyzed by confocal microscopy. Images of the cell surface are used to quantify the fluorescence signal corresponding to the amount of CFTR at the membrane (**Figure 4A**; see Materials and methods). We used treatment with HGF as a positive control, since this growth factor was previously shown to induce the retention of VX-809-rescued F508del CFTR at the cell surface (Moniz et al., 2013; Matos et al., 2018). Indeed, co-treatment with HGF not only increased the overall PM abundance of CFTR at 30°C, as it was able to promote the surface retention of ∼80% of the rescued protein, upon switching to 37°C (**Figure 4B and C**), corresponding to ∼70% of the wt protein at this temperature (**Figure 4C**). Importantly, the knockdown of GABARAP also partially prevented the internalization of rescued F508del-CFTR (**Figure 4B and C**). Albeit having a more moderate effect than HGF, the absence of GABARAP caused the retention of ∼46% of the rescued protein at the cell surface at 37°C (∼25% of wt-CFTR), which when compared to the ∼30% control cells (siLUC; ∼17% of wt-CFTR) reflects a ∼1.5-fold increase in surface protein stability, in agreement with the observed extent of functional rescue.

## Discussion

In this work, we identified novel candidate proteins that are involved in the regulation of CFTR membrane traffic and/or its retention at the PM. The results were obtained based on experimental data from independent proteomic studies conducted in the host lab, namely, on PM CFTR levels following phosphorylation by SYK or the activation of EPAC1- or RAC1-mediated signaling. Subsequently, the resulting interactomes were explored by computational network biology to identify novel protein nodes, never described before to interact with or regulate CFTR.

Recently, several authors have proposed network analysis methods to find novel proteins in common (or mediating the interactions) between two related network modules (Silberberg et al., 2017; Garcia-Vaquero et al., 2018). These candidate proteins may contribute to the common phenotypes associated with both modules. Here, we considered the different sets of CFTR interactors as network modules that have in common their potential role in regulating CFTR stability at the PM. To seek for novel proteins in common between these modules, we selected proteins specifically enriched in direct neighbors from at least two of the CFTR interactor sets. An analogous strategy has been successfully employed to expand the composition of disease-associated network modules (Ghiassian et al., 2015). This method requires candidates to have known direct interactions with module members, so it is limited by the completeness and accuracy of the accessed interaction databases. Our analysis is restricted to physical protein interactions, which will also limit our ability to find candidates regulating CFTR stability through indirect mechanisms such as expression regulation.

Besides the enrichment in protein localization and modification processes, the list of candidate proteins identified 12 (AHSA1, BAG3, COPS5, HDAC6, HSP90AB1, HSP90B1, HSPA5, LGLALS3BP, PABPC1, RACK1, USP19, and YBX1) out of 25 recently reviewed putative CFTR-interacting targets for restoring CFTR biogenesis and function identified through proteomic approaches (Lim et al., 2017). Both observations support the validity of our approach.

Among the novel protein nodes identified, the proteins encoded by the *GABARAP*, *NOS2*, and *SMURF1* genes were functionally validated in the physiologically relevant bronchio-epithelial cell line CFBE. For this, the expression of the endogenous proteins was depleted by RNA interference and the effect on PM localization and function of CFTR was analyzed.

NOS2 encodes the inducible isoform of nitric oxide synthase (NOS), which is expressed maximally following an inflammatory stimulus and produces relatively large micromolar quantities of NO. While macrophages and neutrophils express higher levels of iNOS in the lung, it can also be expressed by bronchial epithelial cells. NOS2 expression in these cells is upregulated by lipopolysaccharide (LPS) and by proinflammatory cytokines, such as interleukin-1, tumor necrosis factor alpha, and gamma interferon (Ermert et al., 2002). Expression of iNOS appears to be nearly absent in bronchial cells from CF patients (Meng et al., 1998; Thomas et al., 2000; Dotsch et al., 2002) and may be caused by abnormalities in the signaling system that normally causes its induction, such as cytokine receptors, second messengers, or transcription factors (Meng et al., 1998; Satitsri et al., 2016). Given the apparent negative functional effect on both wt and VX-809-rescued F508del-CFTR observed here upon iNOS downregulation, our results suggest that, besides an improved resistance to microbial infection, any therapeutic approach that increased iNOS production in the airways might further benefit CF patients by stimulating residual CFTR function. This has, however, to be further investigated since, in a previous study, micromolar concentrations of NO were shown to inhibit forskolin-stimulated cAMP production by adenylyl cyclase and cAMP-dependent Cl^−^ transport and fluid secretion mediated by CFTR (Spirli et al., 2003).

SMURF1 functions as an E3 ubiquitin ligase protein and has been shown previously to regulate endocytosis of other membrane proteins such as the bone morphogenetic protein type II receptor (BMPR), related to pulmonary arterial hypertension. Blockage of SMURF1 ubiquitin ligase activity increased cell surface expression of BMPR (Murakami and Etlinger, 2019). Here, we observed that depletion of SMURF1 produced a twofold stimulation of the iodide influx rate in CFTR wt cells but did not promote any significant rescue of F508del-CFTR to the PM. Moreover, while endogenous cAMP depletion by pretreatment with indomethacin delayed the response to Fsk in control wt-CFTR CFBE cells, this effect was much less pronounced upon SMURF1 knockdown. This is suggestive that the knockdown of these proteins can have an effect not only in CFTR’s PM abundance but also on its gating efficiency. Such an effect might nevertheless have therapeutic potential for class III to V CFTR mutations.

Similar effects were also observed upon knockdown of the GABA receptor-associated protein GABARAP, which belongs to the Atg8 family of ubiquitin-like proteins that are involved in the regulation of autophagy. GABARAP proteins associate with the membrane of the autophagosome through conjugation of phosphatidylethanolamine (Ichimura et al., 2000) and are primary drivers of autophagy working upstream of LC3 proteins and operate mainly at the late stages of autophagy driving the fusion of autophagosomes to lysosomes (Nguyen et al., 2016). Our results show that knockdown of GABARAP increased the temperature-dependent stability and function at the PM of F508del-CFTR after correction with VX-809. Furthermore, depletion of GABARAP also increased wt-CFTR function and, similar to SMURF1 knockdown, its response to Fsk stimulation after pretreatment with indomethacin. Since we observed no detectable impact of GABARAP downregulation on wt-CFTR traffic, it is possible that GABARAP may function by stabilizing the molecular machinery required to anchor and activate CFTR at the PM, thus increasing its gating efficiency. While the high levels of wt-CFTR at the surface may have led to an underestimation of fluorescence signals at the cell surface, the PM stabilizing effects observed with VX-809-rescued F508del-CFTR seem to support a functional potentiation hypothesis. In this case, interference with GABARAP activity may also prove beneficial for class III to class V CFTR mutations.

The identification of novel regulators of CFTR levels at the PM has important biomedical implications. The pharmacological rescue of mutant CFTR folding or gating improves symptoms in CF patients (Zhang et al., 2016); however, a truly relevant clinical benefit appears to require the targeting of additional cellular processes: the trafficking of CFTR as well as its retention at the PM (Farinha and Matos, 2016). The proteins identified here suggest that the cellular processes of membrane traffic, protein stabilization at the PM, and NO signaling are suitable targets for novel therapeutic approaches in CF.

Besides providing new insights into CFTR biology and suggesting possible therapeutic targets in CF, the network biology approach delineated in this work provides a framework applicable to other human diseases related to the traffic and function of PM transport proteins.

## Data Availability Statement

All datasets generated for this study are included in the manuscript and/or the supplementary files.

## Author Contributions

CL, JS, and AM conducted the experiments. PJ, CF, and PM designed the experimental studies. FP provided the network analysis. PJ, CF, PM, and FP analyzed the data and wrote the manuscript.

## Funding

This work was supported by FCT, Portugal, through center grant UID/MULTI/04046/2019 to BioISI and the BioSys PhD program PD65-2012 (fellowships SFRH/BD/52488/2014, SFRH/BD/106084/2015, and SFRH/BD/52490/2014 to CL, JS, and AM, respectively).

## Conflict of Interest Statement

The authors declare that the research was conducted in the absence of any commercial or financial relationships that could be construed as a potential conflict of interest.

## Footnotes

1 CellProfiler, http://cellprofiler.org/ (last accessed October 19, 2017).

2 R program, https://www.r-project.org/ (last accessed March 09, 2018).

## Supplementary Material

Figure S1 qPCR assessment of siRNA efficiency in CFBE cells for the targeted transcript, **p*< 0.05.

Table S1 Specific common neighbours of CFTR interactor sets.

Table S2 Enriched biological processes in the list of strong mediator proteins.
