# Supplementary material for: Network Biology Identifies Novel Regulators of CFTR Trafficking and Membrane Stability
Source: Front Pharmacol. 2019 Jun 4;10:619. doi: 10.3389/fphar.2019.00619 (PMC6559121; doi:10.3389/fphar.2019.00619)

**Network biology identifies novel regulators of CFTR trafficking and membrane stability**

Cláudia Almeida Loureiro*[,1,2], João D Santos*[,1,3], Ana Margarida Matos*[,1,2], Peter Jordan[#,1,2], Paulo Matos[#,1,2], Carlos M Farinha[#,1,3], Francisco R Pinto[1,3]

**Figure S1.** qPCR assessment of siRNA efficiency in CFBE cells for the targeted transcript, * $p < 0.05$

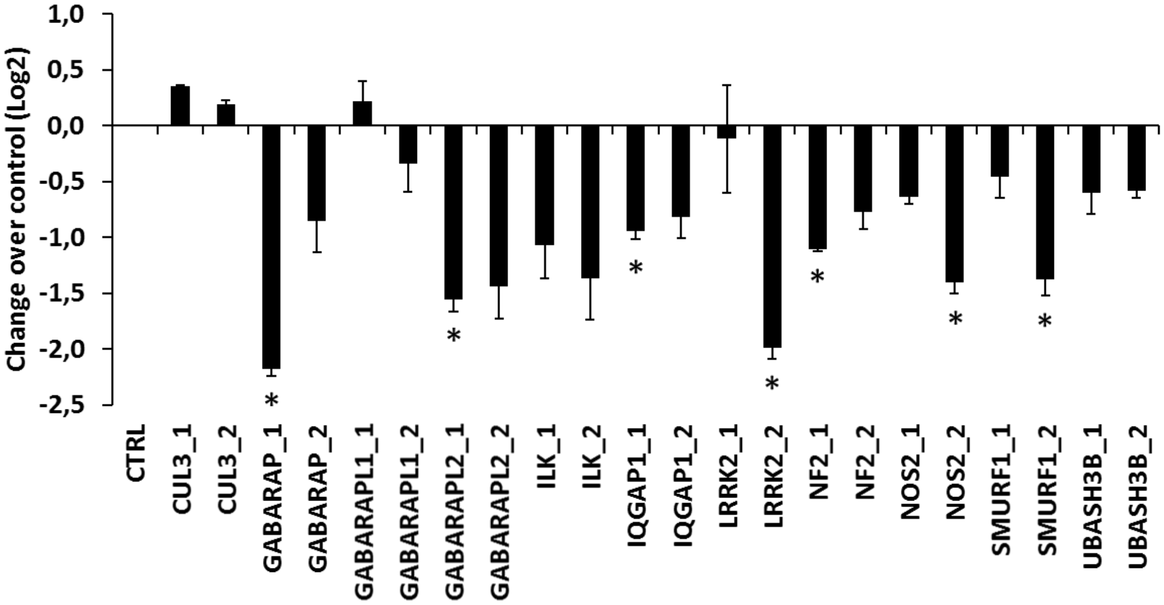

Supplement: Figure S1 — qPCR assessment of siRNA efficiency in CFBE cells for the targeted transcript, *p< 0.05. [file DataSheet_1.pdf]
